# 1-{2-[(Anthracen-10-yl)methyl­ene­amino]phen­yl}-3-phenyl­thio­urea

**DOI:** 10.1107/S1600536807068729

**Published:** 2008-01-09

**Authors:** D. Gayathri, D. Velmurugan, K. Ravikumar, S. Devaraj, M. Kandaswamy

**Affiliations:** a Centre of Advanced Study in Crystallography and Biophysics, University of Madras, Guindy Campus, Chennai 600 025, India; b Laboratory of X-ray Crystallography, Indian Institute of Chemical Technology, Hyderabad 500 007, India; c Department of Inorganic Chemistry, University of Madras, Guindy Campus, Chennai 600 025, India

## Abstract

The title compound, C_28_H_21_N_3_S, crystallizes with two mol­ecules in the asymmetric unit. There are only very slight differences in the torsion angles between the two molecules. The two mol­ecules are stabilized by intra­molecular N—H⋯N inter­actions and the crystal packing is stabilized by inter­molecular N—H⋯S inter­actions.

## Related literature

For related literature, see: Lee *et al.* (2003 ▶, 2005 ▶); Gayathri *et al.* (2007 ▶); Yoon *et al.* (2004 ▶).
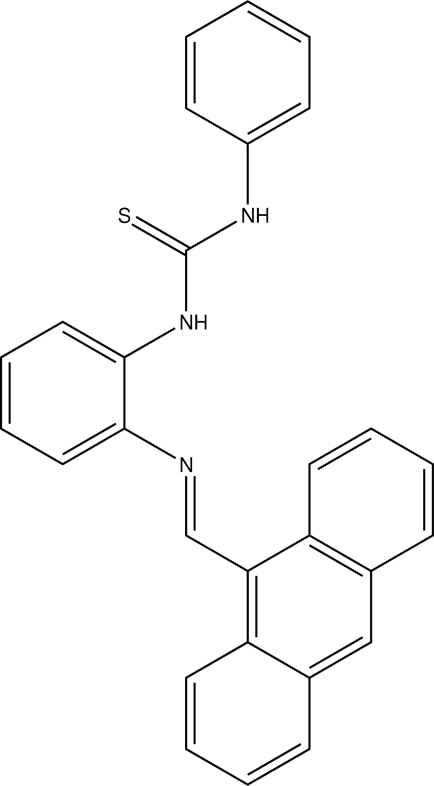


## Experimental

### 

#### Crystal data

C_28_H_21_N_3_S
*M* *_r_* = 431.54
Triclinic, 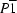

*a* = 6.6148 (6) Å
*b* = 14.3154 (12) Å
*c* = 23.823 (2) Å
α = 74.045 (1)°
β = 88.705 (1)°
γ = 87.421 (1)°
*V* = 2166.7 (3) Å^3^
*Z* = 4
Mo *K*α radiation
μ = 0.17 mm^−1^
*T* = 293 (2) K
0.27 × 0.25 × 0.20 mm

#### Data collection

Bruker SMART APEX CCD area-detector diffractometer
Absorption correction: none
25075 measured reflections
9927 independent reflections
7972 reflections with *I* > 2σ(*I*)
*R*_int_ = 0.019

#### Refinement

*R*[*F* ^2^ > 2σ(*F* ^2^)] = 0.047
*wR*(*F* ^2^) = 0.133
*S* = 1.01
9927 reflections
577 parameters
H-atom parameters constrained
Δρ_max_ = 0.30 e Å^−3^
Δρ_min_ = −0.18 e Å^−3^

### 

Data collection: *SMART* (Bruker, 2001 ▶); cell refinement: *SAINT* (Bruker, 2001 ▶); data reduction: *SAINT*; program(s) used to solve structure: *SHELXS97* (Sheldrick, 2008 ▶); program(s) used to refine structure: *SHELXL97* (Sheldrick, 2008 ▶); molecular graphics: *PLATON* (Spek, 2003 ▶); software used to prepare material for publication: *SHELXL97* and *PARST* (Nardelli, 1995 ▶).

## Supplementary Material

Crystal structure: contains datablocks I, global. DOI: 10.1107/S1600536807068729/at2524sup1.cif

Structure factors: contains datablocks I. DOI: 10.1107/S1600536807068729/at2524Isup2.hkl

Additional supplementary materials:  crystallographic information; 3D view; checkCIF report

## Figures and Tables

**Table 1:** Hydrogen-bond geometry (Å, °)

*D*—H⋯*A*	*D*—H	H⋯*A*	*D*⋯*A*	*D*—H⋯*A*
N1—H1*A*⋯S1^i^	0.86	2.55	3.399 (2)	169
N2—H2*A*⋯N3	0.86	2.22	2.650 (2)	111
N4—H4*A*⋯S2^ii^	0.86	2.63	3.475 (1)	169
N5—H5*A*⋯N6	0.86	2.25	2.666 (2)	110

Symmetry codes: (i) 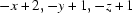; (ii) 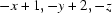.

**Fig. 1.**
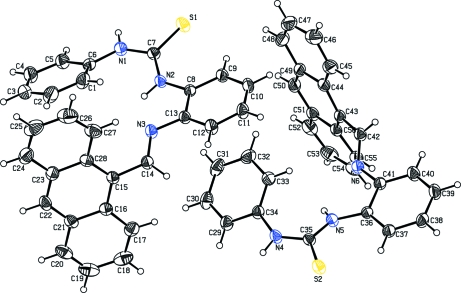
The molecular structure of title compound, showing 30% probability displacement ellipsoids.

**Fig. 2.**
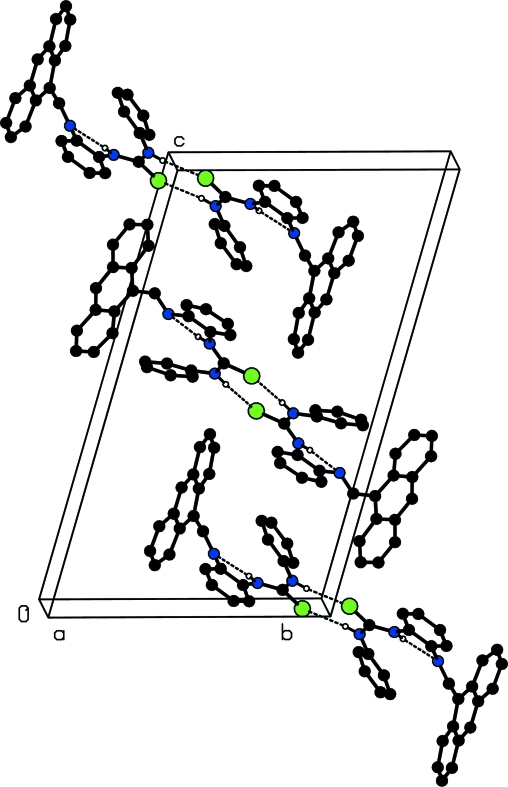
The molecular packing of (I), viewed down the a axis.

## supplementary crystallographic information

### Comment

The design and synthesis of anion receptors possessing high affinity and selectivity represents a challenge that continues to attract considerable attention within the molecular recognition and supramolecular chemistry communities due to the important role anions play in areas as diverse as the environment and medicine (Lee *et al.*, 2005). Of particular interest in this regard are colorimetric anion sensors and fluoresecent chemosensors for anions. Recent efforts to investigate anion binding by naked eye detection (Lee *et al.*, 2003) and through flouresencent changes (Yoon *et al.*, 2004) may provide important results. The most desirable property of an anion sensor based on fluoresecence is the ability to respond to applied perturbation in a highly selective and sensitive manner by dramatic change in emission colour and/or intensity.

The title compound crystallizes in triclinic system with two molecules in asymmetric unit. The bond lengths, bond angles and torsion angles of the title compound are comparable with the similar structure solved earlier (Gayathri *et al.*, 2007). The dihedral angle between the phenyl rings C1–C6 and C8–C13 in molecule A and between C29–C34 and C36–C41 in molecule B are 18.3 (1) and 20.0 (1)°, respectively. The acenapthene moiety in both the molecules are planar as analysed based on the dihedral angles between the rings in the acenapthene moiety.

The two molecules are stabilized by N—H···N intramolecular interactions generating S(5) motif. The crystal packing is stabilized by N—H···S intermolecular interactions generating centrosymmetric dimer of *R*_2_^2^(8) ring.

### Experimental

The compound 1-(2-((anthracene-10-yl)methyleneamino)phenyl)-3-phenylthiourea was synthesized by Schiff base condensation between1-(2-aminophenyl)-3-phenyl thiourea and 9-anthraldehyde. To the solution of 1-(2-aminophenyl)-3-phenyl thiourea (0.5 g, 2.05 mmol) in methanol (25 ml), 4-nitrobenzaldehyde (0.424 g, 2.05 mmol) in methanol was added under stirring. The resulting mixture was heated at reflux for 3 h and cooled to room temperature. The solid product was collected by filtration and washed with cold methanol. The microcrystalline compound was recrystallized from hot acetonitrile; yellow coloured crystals suitable for *X* ray diffraction were obtained on slow evaporation [yield 82.9%,; m.p.: 453 K]

### Refinement

All H-atoms were refined using a riding model with C—H = 0.93Å and N—H = 0.86 Å with *U*_iso_(H)=1.2*U*_eq_ (C,*N*).

### Crystal data

**Table:**

C_28_H_21_N_3_S	*Z* = 4
*M**_r_* = 431.54	*F*_000_ = 904
Triclinic, *P*1	*D*_x_ = 1.323 Mg m^−^^3^
Hall symbol: -P 1	Mo *K*α radiation λ = 0.71073 Å
*a* = 6.6148 (6) Å	Cell parameters from 4967 reflections
*b* = 14.3154 (12) Å	θ = 0.9–25.0º
*c* = 23.823 (2) Å	µ = 0.17 mm^−^^1^
α = 74.045 (1)º	*T* = 293 (2) K
β = 88.705 (1)º	Block, orange
γ = 87.421 (1)º	0.27 × 0.25 × 0.20 mm
*V* = 2166.7 (3) Å^3^	

### Data collection

**Table:**

Bruker SMART APEX CCD area-detector diffractometer	7972 reflections with *I* > 2σ(*I*)
Radiation source: fine-focus sealed tube	*R*_int_ = 0.019
Monochromator: graphite	θ_max_ = 28.0º
*T* = 293(2) K	θ_min_ = 0.9º
ω scans	*h* = −8→8
Absorption correction: none	*k* = −18→18
25075 measured reflections	*l* = −31→30
9927 independent reflections	

### Refinement

**Table:**

Refinement on *F*^2^	Secondary atom site location: difference Fourier map
Least-squares matrix: full	Hydrogen site location: inferred from neighbouring sites
*R*[*F*^2^ > 2σ(*F*^2^)] = 0.047	H-atom parameters constrained
*wR*(*F*^2^) = 0.133	*w* = 1/[σ^2^(*F*_o_^2^) + (0.0719*P*)^2^ + 0.4912*P*] where *P* = (*F*_o_^2^ + 2*F*_c_^2^)/3
*S* = 1.01	(Δ/σ)_max_ = 0.001
9927 reflections	Δρ_max_ = 0.30 e Å^−^^3^
577 parameters	Δρ_min_ = −0.18 e Å^−^^3^
Primary atom site location: structure-invariant direct methods	Extinction correction: none

### Special details

**Table:**

Geometry. All e.s.d.'s (except the e.s.d. in the dihedral angle between two l.s. planes) are estimated using the full covariance matrix. The cell e.s.d.'s are taken into account individually in the estimation of e.s.d.'s in distances, angles and torsion angles; correlations between e.s.d.'s in cell parameters are only used when they are defined by crystal symmetry. An approximate (isotropic) treatment of cell e.s.d.'s is used for estimating e.s.d.'s involving l.s. planes.
Refinement. Refinement of *F*^2^ against ALL reflections. The weighted *R*-factor *wR* and goodness of fit *S* are based on *F*^2^, conventional *R*-factors *R* are based on *F*, with *F* set to zero for negative *F*^2^. The threshold expression of *F*^2^ > σ(*F*^2^) is used only for calculating *R*-factors(gt) *etc*. and is not relevant to the choice of reflections for refinement. *R*-factors based on *F*^2^ are statistically about twice as large as those based on *F*, and *R*- factors based on ALL data will be even larger.

### Fractional atomic coordinates and isotropic or equivalent isotropic displacement parameters (Å^2^)

**Table:**

	*x*	*y*	*z*	*U*_iso_*/*U*_eq_	
C1	1.0750 (3)	0.83551 (13)	0.43543 (8)	0.0668 (5)	
H1	0.9428	0.8512	0.4226	0.080*	
C2	1.2051 (4)	0.90827 (15)	0.43618 (9)	0.0824 (7)	
H2	1.1604	0.9730	0.4227	0.099*	
C3	1.4005 (4)	0.88623 (17)	0.45663 (9)	0.0757 (6)	
H3	1.4874	0.9357	0.4563	0.091*	
C4	1.4646 (3)	0.79053 (17)	0.47747 (9)	0.0661 (5)	
H4	1.5937	0.7750	0.4928	0.079*	
C5	1.3389 (3)	0.71766 (14)	0.47575 (8)	0.0549 (4)	
H5	1.3847	0.6531	0.4893	0.066*	
C6	1.1435 (2)	0.73914 (12)	0.45391 (6)	0.0453 (3)	
C7	0.8606 (2)	0.64513 (10)	0.42670 (7)	0.0407 (3)	
C8	0.6251 (2)	0.73651 (10)	0.34664 (6)	0.0388 (3)	
C9	0.4771 (3)	0.67024 (12)	0.34774 (7)	0.0499 (4)	
H9	0.4953	0.6061	0.3700	0.060*	
C10	0.3022 (3)	0.69976 (13)	0.31565 (8)	0.0533 (4)	
H10	0.2026	0.6553	0.3172	0.064*	
C11	0.2736 (3)	0.79363 (13)	0.28152 (8)	0.0548 (4)	
H11	0.1544	0.8129	0.2608	0.066*	
C12	0.4229 (3)	0.85882 (12)	0.27830 (8)	0.0529 (4)	
H12	0.4054	0.9219	0.2544	0.063*	
C13	0.5993 (2)	0.83184 (10)	0.31016 (7)	0.0405 (3)	
C14	0.7895 (2)	0.96526 (11)	0.26658 (7)	0.0455 (4)	
H14	0.7202	0.9697	0.2323	0.055*	
C15	0.9408 (2)	1.03762 (10)	0.26697 (7)	0.0414 (3)	
C16	1.0866 (2)	1.05835 (10)	0.22175 (7)	0.0428 (3)	
C17	1.0824 (3)	1.02141 (12)	0.17182 (8)	0.0554 (4)	
H17	0.9750	0.9845	0.1673	0.067*	
C18	1.2331 (4)	1.03941 (15)	0.13084 (9)	0.0704 (6)	
H18	1.2263	1.0153	0.0985	0.084*	
C19	1.3993 (4)	1.09424 (16)	0.13679 (10)	0.0738 (6)	
H19	1.5029	1.1044	0.1090	0.089*	
C20	1.4083 (3)	1.13178 (13)	0.18254 (9)	0.0627 (5)	
H20	1.5178	1.1685	0.1857	0.075*	
C21	1.2538 (2)	1.11652 (11)	0.22630 (7)	0.0473 (4)	
C22	1.2633 (3)	1.15498 (11)	0.27368 (8)	0.0527 (4)	
H22	1.3740	1.1908	0.2770	0.063*	
C23	1.1118 (3)	1.14130 (11)	0.31620 (7)	0.0512 (4)	
C24	1.1149 (4)	1.18661 (14)	0.36275 (9)	0.0726 (6)	
H24	1.2239	1.2235	0.3661	0.087*	
C25	0.9601 (5)	1.17627 (17)	0.40198 (10)	0.0892 (8)	
H25	0.9639	1.2060	0.4321	0.107*	
C26	0.7947 (5)	1.12125 (17)	0.39754 (10)	0.0862 (7)	
H26	0.6877	1.1167	0.4240	0.103*	
C27	0.7874 (3)	1.07436 (14)	0.35527 (9)	0.0640 (5)	
H27	0.6774	1.0369	0.3539	0.077*	
C28	0.9462 (3)	1.08186 (11)	0.31304 (7)	0.0464 (4)	
C29	0.8250 (2)	0.82162 (12)	0.11334 (8)	0.0498 (4)	
H29	0.8800	0.8707	0.0836	0.060*	
C30	0.9403 (3)	0.77475 (14)	0.16104 (9)	0.0596 (5)	
H30	1.0722	0.7931	0.1636	0.071*	
C31	0.8624 (3)	0.70121 (14)	0.20488 (9)	0.0660 (5)	
H31	0.9413	0.6693	0.2368	0.079*	
C32	0.6657 (3)	0.67491 (14)	0.20117 (8)	0.0663 (5)	
H32	0.6130	0.6246	0.2306	0.080*	
C33	0.5464 (3)	0.72254 (13)	0.15422 (7)	0.0564 (4)	
H33	0.4130	0.7056	0.1526	0.068*	
C34	0.6266 (2)	0.79581 (11)	0.10948 (7)	0.0423 (3)	
C35	0.3493 (2)	0.83579 (10)	0.03364 (6)	0.0393 (3)	
C36	0.1103 (2)	0.70265 (10)	0.03509 (6)	0.0372 (3)	
C37	−0.0307 (2)	0.74808 (12)	−0.00702 (7)	0.0469 (4)	
H37	−0.0083	0.8100	−0.0311	0.056*	
C38	−0.2040 (3)	0.70172 (13)	−0.01336 (8)	0.0531 (4)	
H38	−0.2979	0.7331	−0.0415	0.064*	
C39	−0.2395 (3)	0.61000 (14)	0.02130 (8)	0.0615 (5)	
H39	−0.3570	0.5794	0.0170	0.074*	
C40	−0.0983 (3)	0.56365 (13)	0.06269 (8)	0.0586 (5)	
H40	−0.1209	0.5011	0.0859	0.070*	
C41	0.0756 (2)	0.60844 (11)	0.07029 (6)	0.0411 (3)	
C42	0.1800 (2)	0.50230 (11)	0.15842 (7)	0.0450 (3)	
H42	0.0437	0.4950	0.1683	0.054*	
C43	0.3335 (2)	0.44540 (10)	0.19903 (7)	0.0427 (3)	
C44	0.3104 (3)	0.43694 (11)	0.25943 (7)	0.0473 (4)	
C45	0.1310 (3)	0.46802 (12)	0.28476 (8)	0.0593 (5)	
H45	0.0216	0.4948	0.2610	0.071*	
C46	0.1176 (4)	0.45915 (14)	0.34297 (9)	0.0736 (6)	
H46	−0.0025	0.4777	0.3587	0.088*	
C47	0.2841 (5)	0.42199 (16)	0.37991 (9)	0.0795 (7)	
H47	0.2748	0.4190	0.4194	0.095*	
C48	0.4553 (4)	0.39112 (15)	0.35819 (8)	0.0699 (6)	
H48	0.5633	0.3669	0.3831	0.084*	
C49	0.4761 (3)	0.39443 (12)	0.29795 (7)	0.0545 (4)	
C50	0.6473 (3)	0.35753 (13)	0.27574 (8)	0.0573 (5)	
H50	0.7555	0.3331	0.3005	0.069*	
C51	0.6633 (2)	0.35574 (11)	0.21783 (7)	0.0500 (4)	
C52	0.8315 (3)	0.30881 (13)	0.19662 (9)	0.0620 (5)	
H52	0.9389	0.2830	0.2214	0.074*	
C53	0.8381 (3)	0.30117 (14)	0.14154 (10)	0.0660 (5)	
H53	0.9487	0.2699	0.1287	0.079*	
C54	0.6768 (3)	0.34067 (13)	0.10326 (9)	0.0587 (4)	
H54	0.6796	0.3329	0.0658	0.070*	
C55	0.5179 (3)	0.38982 (11)	0.12048 (7)	0.0486 (4)	
H55	0.4162	0.4173	0.0940	0.058*	
C56	0.5029 (2)	0.40045 (10)	0.17814 (7)	0.0426 (3)	
N1	1.0247 (2)	0.65807 (9)	0.45631 (6)	0.0500 (3)	
H1A	1.0655	0.6061	0.4820	0.060*	
N2	0.8043 (2)	0.71818 (9)	0.38004 (6)	0.0456 (3)	
H2A	0.8932	0.7616	0.3684	0.055*	
N3	0.75168 (19)	0.89754 (9)	0.31147 (6)	0.0452 (3)	
N4	0.5165 (2)	0.85242 (9)	0.06068 (6)	0.0485 (3)	
H4A	0.5654	0.9084	0.0452	0.058*	
N5	0.29052 (19)	0.74304 (9)	0.04688 (6)	0.0436 (3)	
H5A	0.3786	0.7009	0.0659	0.052*	
N6	0.22933 (19)	0.56134 (9)	0.11000 (6)	0.0435 (3)	
S1	0.74429 (7)	0.53913 (3)	0.45046 (2)	0.05533 (13)	
S2	0.23302 (6)	0.93114 (3)	−0.01346 (2)	0.05342 (13)	

### Atomic displacement parameters (Å^2^)

**Table:**

	*U*^11^	*U*^22^	*U*^33^	*U*^12^	*U*^13^	*U*^23^
C1	0.0887 (14)	0.0436 (9)	0.0634 (11)	−0.0040 (9)	−0.0342 (10)	−0.0039 (8)
C2	0.132 (2)	0.0445 (10)	0.0649 (12)	−0.0186 (12)	−0.0358 (13)	−0.0002 (9)
C3	0.1011 (17)	0.0719 (14)	0.0559 (11)	−0.0431 (13)	−0.0029 (11)	−0.0140 (10)
C4	0.0545 (11)	0.0793 (14)	0.0698 (12)	−0.0173 (10)	0.0013 (9)	−0.0274 (11)
C5	0.0492 (9)	0.0562 (10)	0.0595 (10)	−0.0036 (8)	−0.0024 (8)	−0.0156 (8)
C6	0.0528 (9)	0.0423 (8)	0.0377 (8)	−0.0060 (7)	−0.0069 (6)	−0.0045 (6)
C7	0.0406 (7)	0.0336 (7)	0.0435 (8)	0.0008 (6)	−0.0049 (6)	−0.0032 (6)
C8	0.0415 (7)	0.0345 (7)	0.0373 (7)	−0.0041 (6)	−0.0073 (6)	−0.0038 (6)
C9	0.0583 (10)	0.0373 (8)	0.0486 (9)	−0.0115 (7)	−0.0134 (7)	−0.0002 (7)
C10	0.0507 (9)	0.0484 (9)	0.0589 (10)	−0.0162 (7)	−0.0136 (7)	−0.0083 (8)
C11	0.0455 (9)	0.0490 (9)	0.0672 (11)	−0.0035 (7)	−0.0212 (8)	−0.0092 (8)
C12	0.0500 (9)	0.0354 (8)	0.0672 (11)	−0.0011 (7)	−0.0186 (8)	−0.0025 (7)
C13	0.0406 (7)	0.0329 (7)	0.0457 (8)	−0.0040 (6)	−0.0078 (6)	−0.0055 (6)
C14	0.0452 (8)	0.0364 (7)	0.0498 (9)	−0.0024 (6)	−0.0141 (7)	−0.0021 (6)
C15	0.0448 (8)	0.0279 (6)	0.0455 (8)	−0.0017 (6)	−0.0099 (6)	0.0007 (6)
C16	0.0477 (8)	0.0284 (7)	0.0458 (8)	0.0022 (6)	−0.0096 (6)	0.0009 (6)
C17	0.0681 (11)	0.0430 (9)	0.0516 (10)	0.0030 (8)	−0.0110 (8)	−0.0069 (7)
C18	0.0955 (16)	0.0606 (12)	0.0484 (10)	0.0151 (11)	0.0003 (10)	−0.0065 (9)
C19	0.0757 (14)	0.0616 (12)	0.0665 (13)	0.0092 (11)	0.0179 (11)	0.0087 (10)
C20	0.0554 (10)	0.0448 (9)	0.0731 (13)	−0.0020 (8)	0.0060 (9)	0.0082 (9)
C21	0.0471 (8)	0.0304 (7)	0.0549 (9)	−0.0010 (6)	−0.0060 (7)	0.0046 (6)
C22	0.0544 (9)	0.0330 (7)	0.0645 (10)	−0.0110 (7)	−0.0154 (8)	−0.0003 (7)
C23	0.0677 (11)	0.0318 (7)	0.0502 (9)	−0.0048 (7)	−0.0142 (8)	−0.0034 (7)
C24	0.1128 (18)	0.0435 (10)	0.0621 (12)	−0.0131 (11)	−0.0221 (12)	−0.0124 (9)
C25	0.156 (3)	0.0593 (13)	0.0565 (12)	−0.0048 (15)	−0.0014 (15)	−0.0237 (10)
C26	0.129 (2)	0.0610 (13)	0.0683 (14)	−0.0083 (14)	0.0262 (14)	−0.0183 (11)
C27	0.0812 (13)	0.0460 (10)	0.0611 (11)	−0.0065 (9)	0.0113 (10)	−0.0086 (8)
C28	0.0571 (9)	0.0305 (7)	0.0460 (8)	−0.0021 (6)	−0.0057 (7)	−0.0007 (6)
C29	0.0455 (9)	0.0467 (9)	0.0562 (10)	−0.0021 (7)	−0.0099 (7)	−0.0116 (7)
C30	0.0490 (9)	0.0618 (11)	0.0711 (12)	0.0055 (8)	−0.0229 (8)	−0.0231 (10)
C31	0.0815 (13)	0.0551 (11)	0.0612 (11)	0.0114 (10)	−0.0356 (10)	−0.0150 (9)
C32	0.0912 (15)	0.0551 (11)	0.0465 (10)	−0.0107 (10)	−0.0176 (9)	−0.0009 (8)
C33	0.0576 (10)	0.0574 (10)	0.0483 (9)	−0.0124 (8)	−0.0103 (8)	−0.0025 (8)
C34	0.0426 (8)	0.0373 (7)	0.0453 (8)	0.0005 (6)	−0.0106 (6)	−0.0079 (6)
C35	0.0352 (7)	0.0360 (7)	0.0416 (7)	−0.0021 (6)	−0.0029 (6)	−0.0020 (6)
C36	0.0371 (7)	0.0351 (7)	0.0370 (7)	−0.0019 (6)	−0.0050 (5)	−0.0054 (6)
C37	0.0505 (9)	0.0402 (8)	0.0446 (8)	−0.0025 (7)	−0.0131 (7)	−0.0014 (6)
C38	0.0500 (9)	0.0536 (10)	0.0511 (9)	−0.0028 (7)	−0.0204 (7)	−0.0050 (8)
C39	0.0524 (10)	0.0588 (11)	0.0668 (11)	−0.0167 (8)	−0.0214 (8)	−0.0028 (9)
C40	0.0595 (10)	0.0439 (9)	0.0632 (11)	−0.0145 (8)	−0.0194 (8)	0.0044 (8)
C41	0.0422 (8)	0.0354 (7)	0.0423 (8)	−0.0016 (6)	−0.0088 (6)	−0.0042 (6)
C42	0.0443 (8)	0.0364 (7)	0.0496 (9)	−0.0059 (6)	−0.0089 (7)	−0.0027 (6)
C43	0.0488 (8)	0.0301 (7)	0.0445 (8)	−0.0099 (6)	−0.0111 (6)	−0.0003 (6)
C44	0.0624 (10)	0.0293 (7)	0.0474 (8)	−0.0131 (7)	−0.0077 (7)	−0.0033 (6)
C45	0.0802 (13)	0.0384 (8)	0.0576 (10)	−0.0085 (8)	−0.0003 (9)	−0.0093 (8)
C46	0.1098 (18)	0.0483 (10)	0.0660 (13)	−0.0177 (11)	0.0168 (12)	−0.0202 (9)
C47	0.139 (2)	0.0554 (12)	0.0477 (11)	−0.0326 (13)	−0.0053 (13)	−0.0151 (9)
C48	0.1050 (17)	0.0554 (11)	0.0480 (10)	−0.0246 (11)	−0.0185 (11)	−0.0072 (9)
C49	0.0767 (12)	0.0377 (8)	0.0451 (9)	−0.0212 (8)	−0.0178 (8)	−0.0003 (7)
C50	0.0595 (10)	0.0477 (9)	0.0557 (10)	−0.0130 (8)	−0.0242 (8)	0.0044 (8)
C51	0.0485 (9)	0.0358 (8)	0.0562 (10)	−0.0107 (7)	−0.0150 (7)	0.0060 (7)
C52	0.0464 (9)	0.0461 (9)	0.0779 (13)	−0.0008 (7)	−0.0127 (8)	0.0099 (9)
C53	0.0557 (11)	0.0487 (10)	0.0820 (14)	0.0014 (8)	0.0086 (10)	−0.0001 (9)
C54	0.0654 (11)	0.0454 (9)	0.0600 (11)	−0.0072 (8)	0.0055 (9)	−0.0053 (8)
C55	0.0531 (9)	0.0383 (8)	0.0486 (9)	−0.0064 (7)	−0.0064 (7)	−0.0011 (7)
C56	0.0452 (8)	0.0287 (7)	0.0472 (8)	−0.0089 (6)	−0.0111 (6)	0.0027 (6)
N1	0.0529 (8)	0.0357 (6)	0.0524 (8)	−0.0039 (6)	−0.0181 (6)	0.0048 (6)
N2	0.0462 (7)	0.0360 (6)	0.0461 (7)	−0.0118 (5)	−0.0124 (5)	0.0054 (5)
N3	0.0439 (7)	0.0313 (6)	0.0547 (8)	−0.0045 (5)	−0.0142 (6)	−0.0009 (5)
N4	0.0442 (7)	0.0377 (7)	0.0549 (8)	−0.0094 (5)	−0.0144 (6)	0.0042 (6)
N5	0.0390 (6)	0.0337 (6)	0.0508 (7)	−0.0011 (5)	−0.0137 (5)	0.0015 (5)
N6	0.0452 (7)	0.0324 (6)	0.0471 (7)	−0.0041 (5)	−0.0120 (5)	0.0001 (5)
S1	0.0498 (2)	0.0344 (2)	0.0690 (3)	−0.00536 (16)	−0.01566 (19)	0.00915 (18)
S2	0.0460 (2)	0.0368 (2)	0.0651 (3)	−0.00503 (16)	−0.01748 (18)	0.00861 (18)

### Geometric parameters (Å, °)

**Table:**

C1—C2	1.385 (3)	C29—H29	0.9300
C1—C6	1.386 (2)	C30—C31	1.373 (3)
C1—H1	0.9300	C30—H30	0.9300
C2—C3	1.384 (3)	C31—C32	1.382 (3)
C2—H2	0.9300	C31—H31	0.9300
C3—C4	1.373 (3)	C32—C33	1.382 (2)
C3—H3	0.9300	C32—H32	0.9300
C4—C5	1.373 (3)	C33—C34	1.388 (2)
C4—H4	0.9300	C33—H33	0.9300
C5—C6	1.393 (2)	C34—N4	1.4164 (18)
C5—H5	0.9300	C35—N5	1.3506 (19)
C6—N1	1.418 (2)	C35—N4	1.3545 (19)
C7—N2	1.3462 (18)	C35—S2	1.6782 (14)
C7—N1	1.354 (2)	C36—C37	1.3884 (19)
C7—S1	1.6818 (15)	C36—C41	1.404 (2)
C8—C9	1.389 (2)	C36—N5	1.4146 (18)
C8—C13	1.4073 (19)	C37—C38	1.382 (2)
C8—N2	1.4164 (18)	C37—H37	0.9300
C9—C10	1.385 (2)	C38—C39	1.373 (2)
C9—H9	0.9300	C38—H38	0.9300
C10—C11	1.373 (2)	C39—C40	1.382 (2)
C10—H10	0.9300	C39—H39	0.9300
C11—C12	1.376 (2)	C40—C41	1.381 (2)
C11—H11	0.9300	C40—H40	0.9300
C12—C13	1.387 (2)	C41—N6	1.4192 (18)
C12—H12	0.9300	C42—N6	1.274 (2)
C13—N3	1.4159 (18)	C42—C43	1.472 (2)
C14—N3	1.259 (2)	C42—H42	0.9300
C14—C15	1.475 (2)	C43—C56	1.415 (2)
C14—H14	0.9300	C43—C44	1.416 (2)
C15—C16	1.408 (2)	C44—C45	1.427 (3)
C15—C28	1.412 (2)	C44—C49	1.446 (2)
C16—C17	1.430 (2)	C45—C46	1.359 (3)
C16—C21	1.437 (2)	C45—H45	0.9300
C17—C18	1.361 (3)	C46—C47	1.416 (3)
C17—H17	0.9300	C46—H46	0.9300
C18—C19	1.411 (3)	C47—C48	1.341 (3)
C18—H18	0.9300	C47—H47	0.9300
C19—C20	1.345 (3)	C48—C49	1.427 (3)
C19—H19	0.9300	C48—H48	0.9300
C20—C21	1.423 (3)	C49—C50	1.384 (3)
C20—H20	0.9300	C50—C51	1.388 (3)
C21—C22	1.389 (3)	C50—H50	0.9300
C22—C23	1.390 (3)	C51—C52	1.428 (3)
C22—H22	0.9300	C51—C56	1.443 (2)
C23—C24	1.431 (3)	C52—C53	1.346 (3)
C23—C28	1.432 (2)	C52—H52	0.9300
C24—C25	1.356 (4)	C53—C54	1.412 (3)
C24—H24	0.9300	C53—H53	0.9300
C25—C26	1.399 (4)	C54—C55	1.357 (3)
C25—H25	0.9300	C54—H54	0.9300
C26—C27	1.358 (3)	C55—C56	1.424 (2)
C26—H26	0.9300	C55—H55	0.9300
C27—C28	1.425 (3)	N1—H1A	0.8600
C27—H27	0.9300	N2—H2A	0.8600
C29—C30	1.377 (2)	N4—H4A	0.8600
C29—C34	1.391 (2)	N5—H5A	0.8600
			
C2—C1—C6	119.44 (19)	C30—C31—H31	120.3
C2—C1—H1	120.3	C32—C31—H31	120.3
C6—C1—H1	120.3	C31—C32—C33	120.71 (18)
C3—C2—C1	121.1 (2)	C31—C32—H32	119.6
C3—C2—H2	119.5	C33—C32—H32	119.6
C1—C2—H2	119.5	C32—C33—C34	119.71 (17)
C4—C3—C2	119.27 (19)	C32—C33—H33	120.1
C4—C3—H3	120.4	C34—C33—H33	120.1
C2—C3—H3	120.4	C33—C34—C29	119.27 (15)
C5—C4—C3	120.3 (2)	C33—C34—N4	124.68 (14)
C5—C4—H4	119.9	C29—C34—N4	115.92 (14)
C3—C4—H4	119.9	N5—C35—N4	116.82 (13)
C4—C5—C6	120.85 (18)	N5—C35—S2	125.39 (11)
C4—C5—H5	119.6	N4—C35—S2	117.79 (11)
C6—C5—H5	119.6	C37—C36—C41	119.01 (13)
C1—C6—C5	118.99 (16)	C37—C36—N5	125.81 (13)
C1—C6—N1	124.94 (16)	C41—C36—N5	115.19 (12)
C5—C6—N1	115.92 (14)	C38—C37—C36	120.32 (14)
N2—C7—N1	117.21 (13)	C38—C37—H37	119.8
N2—C7—S1	125.02 (12)	C36—C37—H37	119.8
N1—C7—S1	117.76 (11)	C39—C38—C37	120.89 (15)
C9—C8—C13	118.98 (13)	C39—C38—H38	119.6
C9—C8—N2	126.00 (13)	C37—C38—H38	119.6
C13—C8—N2	115.01 (12)	C38—C39—C40	119.14 (16)
C10—C9—C8	119.98 (14)	C38—C39—H39	120.4
C10—C9—H9	120.0	C40—C39—H39	120.4
C8—C9—H9	120.0	C41—C40—C39	121.25 (16)
C11—C10—C9	121.11 (15)	C41—C40—H40	119.4
C11—C10—H10	119.4	C39—C40—H40	119.4
C9—C10—H10	119.4	C40—C41—C36	119.38 (13)
C10—C11—C12	119.34 (15)	C40—C41—N6	122.91 (13)
C10—C11—H11	120.3	C36—C41—N6	117.60 (13)
C12—C11—H11	120.3	N6—C42—C43	121.60 (15)
C11—C12—C13	121.02 (15)	N6—C42—H42	119.2
C11—C12—H12	119.5	C43—C42—H42	119.2
C13—C12—H12	119.5	C56—C43—C44	120.31 (14)
C12—C13—C8	119.47 (13)	C56—C43—C42	120.68 (14)
C12—C13—N3	123.33 (13)	C44—C43—C42	119.00 (15)
C8—C13—N3	117.03 (12)	C43—C44—C45	123.47 (16)
N3—C14—C15	121.62 (14)	C43—C44—C49	118.77 (16)
N3—C14—H14	119.2	C45—C44—C49	117.76 (16)
C15—C14—H14	119.2	C46—C45—C44	121.1 (2)
C16—C15—C28	120.45 (14)	C46—C45—H45	119.5
C16—C15—C14	118.51 (14)	C44—C45—H45	119.5
C28—C15—C14	120.97 (14)	C45—C46—C47	120.8 (2)
C15—C16—C17	123.43 (15)	C45—C46—H46	119.6
C15—C16—C21	119.14 (15)	C47—C46—H46	119.6
C17—C16—C21	117.39 (15)	C48—C47—C46	120.2 (2)
C18—C17—C16	121.09 (18)	C48—C47—H47	119.9
C18—C17—H17	119.5	C46—C47—H47	119.9
C16—C17—H17	119.5	C47—C48—C49	121.7 (2)
C17—C18—C19	120.9 (2)	C47—C48—H48	119.1
C17—C18—H18	119.6	C49—C48—H48	119.1
C19—C18—H18	119.6	C50—C49—C48	122.22 (18)
C20—C19—C18	120.17 (19)	C50—C49—C44	119.51 (15)
C20—C19—H19	119.9	C48—C49—C44	118.3 (2)
C18—C19—H19	119.9	C49—C50—C51	122.32 (15)
C19—C20—C21	121.42 (19)	C49—C50—H50	118.8
C19—C20—H20	119.3	C51—C50—H50	118.8
C21—C20—H20	119.3	C50—C51—C52	121.97 (16)
C22—C21—C20	121.45 (17)	C50—C51—C56	119.13 (17)
C22—C21—C16	119.52 (15)	C52—C51—C56	118.88 (16)
C20—C21—C16	119.02 (17)	C53—C52—C51	121.42 (17)
C21—C22—C23	121.73 (15)	C53—C52—H52	119.3
C21—C22—H22	119.1	C51—C52—H52	119.3
C23—C22—H22	119.1	C52—C53—C54	119.96 (18)
C22—C23—C24	121.61 (18)	C52—C53—H53	120.0
C22—C23—C28	119.49 (15)	C54—C53—H53	120.0
C24—C23—C28	118.89 (18)	C55—C54—C53	120.86 (19)
C25—C24—C23	120.5 (2)	C55—C54—H54	119.6
C25—C24—H24	119.7	C53—C54—H54	119.6
C23—C24—H24	119.7	C54—C55—C56	121.67 (16)
C24—C25—C26	120.5 (2)	C54—C55—H55	119.2
C24—C25—H25	119.7	C56—C55—H55	119.2
C26—C25—H25	119.7	C43—C56—C55	123.44 (14)
C27—C26—C25	121.2 (2)	C43—C56—C51	119.30 (15)
C27—C26—H26	119.4	C55—C56—C51	117.07 (15)
C25—C26—H26	119.4	C7—N1—C6	133.57 (13)
C26—C27—C28	120.8 (2)	C7—N1—H1A	113.2
C26—C27—H27	119.6	C6—N1—H1A	113.2
C28—C27—H27	119.6	C7—N2—C8	131.67 (13)
C15—C28—C27	122.69 (16)	C7—N2—H2A	114.2
C15—C28—C23	119.31 (15)	C8—N2—H2A	114.2
C27—C28—C23	117.91 (16)	C14—N3—C13	120.04 (13)
C30—C29—C34	120.22 (17)	C35—N4—C34	132.70 (13)
C30—C29—H29	119.9	C35—N4—H4A	113.6
C34—C29—H29	119.9	C34—N4—H4A	113.6
C31—C30—C29	120.60 (17)	C35—N5—C36	131.68 (12)
C31—C30—H30	119.7	C35—N5—H5A	114.2
C29—C30—H30	119.7	C36—N5—H5A	114.2
C30—C31—C32	119.46 (17)	C42—N6—C41	119.36 (13)
			
C6—C1—C2—C3	−1.9 (3)	C37—C38—C39—C40	−0.5 (3)
C1—C2—C3—C4	−1.2 (3)	C38—C39—C40—C41	0.9 (3)
C2—C3—C4—C5	2.7 (3)	C39—C40—C41—C36	−0.3 (3)
C3—C4—C5—C6	−1.2 (3)	C39—C40—C41—N6	−176.40 (18)
C2—C1—C6—C5	3.4 (3)	C37—C36—C41—C40	−0.7 (2)
C2—C1—C6—N1	178.75 (19)	N5—C36—C41—C40	178.87 (15)
C4—C5—C6—C1	−1.9 (3)	C37—C36—C41—N6	175.57 (14)
C4—C5—C6—N1	−177.70 (17)	N5—C36—C41—N6	−4.9 (2)
C13—C8—C9—C10	−3.4 (3)	N6—C42—C43—C56	−43.0 (2)
N2—C8—C9—C10	175.63 (16)	N6—C42—C43—C44	136.46 (16)
C8—C9—C10—C11	1.3 (3)	C56—C43—C44—C45	−170.72 (14)
C9—C10—C11—C12	1.3 (3)	C42—C43—C44—C45	9.9 (2)
C10—C11—C12—C13	−1.7 (3)	C56—C43—C44—C49	9.1 (2)
C11—C12—C13—C8	−0.4 (3)	C42—C43—C44—C49	−170.34 (13)
C11—C12—C13—N3	−175.68 (17)	C43—C44—C45—C46	−179.29 (16)
C9—C8—C13—C12	3.0 (2)	C49—C44—C45—C46	0.9 (2)
N2—C8—C13—C12	−176.15 (15)	C44—C45—C46—C47	2.3 (3)
C9—C8—C13—N3	178.55 (15)	C45—C46—C47—C48	−2.8 (3)
N2—C8—C13—N3	−0.6 (2)	C46—C47—C48—C49	0.0 (3)
N3—C14—C15—C16	131.51 (17)	C47—C48—C49—C50	−176.09 (18)
N3—C14—C15—C28	−45.5 (2)	C47—C48—C49—C44	3.2 (3)
C28—C15—C16—C17	−175.64 (14)	C43—C44—C49—C50	−4.1 (2)
C14—C15—C16—C17	7.4 (2)	C45—C44—C49—C50	175.73 (15)
C28—C15—C16—C21	6.8 (2)	C43—C44—C49—C48	176.64 (15)
C14—C15—C16—C21	−170.17 (13)	C45—C44—C49—C48	−3.5 (2)
C15—C16—C17—C18	−176.45 (16)	C48—C49—C50—C51	176.32 (16)
C21—C16—C17—C18	1.1 (2)	C44—C49—C50—C51	−2.9 (2)
C16—C17—C18—C19	0.7 (3)	C49—C50—C51—C52	−173.56 (15)
C17—C18—C19—C20	−1.8 (3)	C49—C50—C51—C56	4.9 (2)
C18—C19—C20—C21	0.8 (3)	C50—C51—C52—C53	174.92 (17)
C19—C20—C21—C22	179.76 (17)	C56—C51—C52—C53	−3.5 (2)
C19—C20—C21—C16	1.0 (3)	C51—C52—C53—C54	0.5 (3)
C15—C16—C21—C22	−3.1 (2)	C52—C53—C54—C55	2.6 (3)
C17—C16—C21—C22	179.26 (14)	C53—C54—C55—C56	−2.4 (3)
C15—C16—C21—C20	175.70 (14)	C44—C43—C56—C55	167.66 (14)
C17—C16—C21—C20	−2.0 (2)	C42—C43—C56—C55	−12.9 (2)
C20—C21—C22—C23	179.24 (15)	C44—C43—C56—C51	−7.2 (2)
C16—C21—C22—C23	−2.0 (2)	C42—C43—C56—C51	172.21 (13)
C21—C22—C23—C24	−175.56 (16)	C54—C55—C56—C43	−175.61 (15)
C21—C22—C23—C28	3.3 (2)	C54—C55—C56—C51	−0.6 (2)
C22—C23—C24—C25	176.57 (19)	C50—C51—C56—C43	0.2 (2)
C28—C23—C24—C25	−2.3 (3)	C52—C51—C56—C43	178.72 (14)
C23—C24—C25—C26	−0.1 (4)	C50—C51—C56—C55	−174.95 (14)
C24—C25—C26—C27	2.2 (4)	C52—C51—C56—C55	3.5 (2)
C25—C26—C27—C28	−1.7 (3)	N2—C7—N1—C6	9.4 (3)
C16—C15—C28—C27	170.93 (15)	S1—C7—N1—C6	−171.10 (15)
C14—C15—C28—C27	−12.2 (2)	C1—C6—N1—C7	26.5 (3)
C16—C15—C28—C23	−5.6 (2)	C5—C6—N1—C7	−157.95 (18)
C14—C15—C28—C23	171.36 (14)	N1—C7—N2—C8	−166.81 (16)
C26—C27—C28—C15	−177.34 (18)	S1—C7—N2—C8	13.8 (3)
C26—C27—C28—C23	−0.8 (3)	C9—C8—N2—C7	−14.2 (3)
C22—C23—C28—C15	0.5 (2)	C13—C8—N2—C7	164.89 (16)
C24—C23—C28—C15	179.40 (15)	C15—C14—N3—C13	177.32 (14)
C22—C23—C28—C27	−176.18 (15)	C12—C13—N3—C14	−34.9 (2)
C24—C23—C28—C27	2.7 (2)	C8—C13—N3—C14	149.78 (16)
C34—C29—C30—C31	1.0 (3)	N5—C35—N4—C34	−14.2 (3)
C29—C30—C31—C32	−0.7 (3)	S2—C35—N4—C34	165.96 (15)
C30—C31—C32—C33	−0.7 (3)	C33—C34—N4—C35	−25.0 (3)
C31—C32—C33—C34	1.8 (3)	C29—C34—N4—C35	159.31 (17)
C32—C33—C34—C29	−1.4 (3)	N4—C35—N5—C36	167.90 (15)
C32—C33—C34—N4	−177.02 (17)	S2—C35—N5—C36	−12.3 (3)
C30—C29—C34—C33	0.1 (3)	C37—C36—N5—C35	18.4 (3)
C30—C29—C34—N4	176.05 (16)	C41—C36—N5—C35	−161.10 (16)
C41—C36—C37—C38	1.1 (2)	C43—C42—N6—C41	174.85 (14)
N5—C36—C37—C38	−178.38 (15)	C40—C41—N6—C42	−36.1 (2)
C36—C37—C38—C39	−0.5 (3)	C36—C41—N6—C42	147.81 (15)

### Hydrogen-bond geometry (Å, °)

**Table:**

*D*—H···*A*	*D*—H	H···*A*	*D*···*A*	*D*—H···*A*
N1—H1A···S1^i^	0.86	2.55	3.399 (2)	169
N2—H2A···N3	0.86	2.22	2.650 (2)	111
N4—H4A···S2^ii^	0.86	2.63	3.475 (1)	169
N5—H5A···N6	0.86	2.25	2.666 (2)	110

Symmetry codes: (i) −*x*+2, −*y*+1, −*z*+1;  (ii) −*x*+1, −*y*+2, −*z*.
